# Salivary Inflammatory Mediator Profiling and Correlation to Clinical Disease Markers in Asthma

**DOI:** 10.1371/journal.pone.0084449

**Published:** 2014-01-07

**Authors:** Frédéric F. Little, Diana M. Delgado, Philip J. Wexler, Frank G. Oppenheim, Patricia Mitchell, James A. Feldman, David R. Walt, Roger D. Peng, Elizabeth C. Matsui

**Affiliations:** 1 Pulmonary Center, Boston University School of Medicine, Boston, Massachusetts, United States of America; 2 Department of Periodontology and Oral Biology, Boston University Goldman School of Dental Medicine, Boston, Massachusetts, United States of America; 3 Department of Emergency Medicine, Boston University School of Medicine, Boston, Massachusetts, United States of America; 4 Department of Chemistry, Tufts University, Medford, Massachusetts, United States of America; 5 Department of Biostatistics, Johns Hopkins Bloomberg School of Public Health, Baltimore, Maryland, United States of America; 6 Division of Pediatric Allergy and Immunology, Johns Hopkins University School of Medicine, Baltimore, Maryland, United States of America; National Heart and Lung institute, United Kingdom

## Abstract

**Rationale:**

There is a need for a readily available, non-invasive source of biomarkers that predict poor asthma control.

**Objectives:**

We sought to determine if there is an association between the salivary inflammatory profile and disease control in children and adults with asthma.

**Methods:**

In this cross-sectional study, we collected demographic and clinical information from two independent populations at different sites, resulting in convenience samples of 58 pediatric and 122 adult urban asthmatics. Control was assessed by symptom questionnaire (children) and by Asthma Control Questionnaire and current exacerbation (adults). Saliva was collected in all subjects. We applied principal component analysis to a 10-plex panel of relevant inflammatory markers to characterize marker profiles and determined if profiles were associated with asthma control.

**Results:**

There were similar, strong correlations amongst biologically related markers in both populations: eosinophil-related: eotaxin-1/CCL11, RANTES/CCL5, and IL-5 (p<.001); myeloid/innate: IL-1β, IL-6, MCP-1/CCL2, and IL-8/CXCL8 (p<.001). The first three principal components captured ≥74% of variability across all ten analytes in both populations. In adults, the Principal Component 1 score, broadly reflective of all markers, but with greater weight given to myeloid/innate markers, was associated with Asthma Control Questionnaire score and exacerbation. The Principal Component 3 score, reflective of IP-10/CXCL10, was associated with current exacerbation. In children, the Principal Component 1, 2, and 3 scores were associated with recent asthma symptoms. The Principal Component 2 score, reflective of higher eosinophil markers, was inversely correlated with symptoms. The Principal Component 3 score was positively associated with all symptom outcomes.

**Conclusion:**

The salivary inflammatory profile is associated with disease control in children and adults with asthma.

## Introduction

Despite marked reductions in asthma morbidity over the past two decades, there continue to be groups of patients with a high burden of asthma morbidity. These groups include patients from certain ethnic minority groups, patients with low socioeconomic status, and patients with severe or brittle asthma [1]–[3]. A biomarker that predicts impending loss of asthma control could be used to monitor high-risk patients and titrate treatment to prevent exacerbations.

While there has been significant progress in applying biomarkers as predictors of clinical disease activity, there remain several limitations of the current biomarkers, both with respect to strength of association with disease activity markers and feasibility of specimen collection. Studies that have employed induced sputum (IS) have focused primarily on adults, as IS is not readily performed in children in a physician office setting. IS, along with bronchoalveolar lavage (BAL) and nasal lavage (NL), require special equipment, are invasive, or depend on technical expertise. Although fractional exhaled nitric oxide (F_E_NO) has received much attention as a promising biomarker, it has proven to be largely a marker of atopy [4] and its utility in clinical decision-making has limitations [5].

Based on the characteristics and limitations of currently available sources of biomarkers and the anatomical connection between the oral cavity and airways, we hypothesized that clinical features of asthma could be reflected in the inflammatory composition of the oral cavity. This hypothesis was supported by several studies showing that biospecimens from sites that are not directly involved in organ-specific diseases can provide useful biomarkers [6]–[8]. Therefore, we explored the utility of measuring inflammatory mediators from whole saliva as a means to assess asthma control. Whole saliva is readily available in virtually all subjects, and non-invasively collected in a few minutes. Our objectives were to 1) measure multiple relevant inflammatory mediators in saliva [9] from two distinct populations of pediatric and adult asthmatics, 2) characterize the asthmatic salivary inflammatory profile, and 3) compare salivary mediator profiles with markers of disease control. Because our hypothesis focused on the salivary inflammatory profile, rather than any individual inflammatory markers, we used Principal Component Analysis (PCA), which is a statistical method that captures key *patterns* of marker profiles from a panel of multiple analytes. We identified consistent patterns of salivary inflammatory markers in children and adults, and identified marker profiles, which correlated with clinical indicators of asthma disease control in both populations.

## Methods

### Study Design and Subject Recruitment

The sample of pediatric study subjects was derived from the Mouse Allergen and Asthma Cohort Study (MAACS), a longitudinal study of one hundred fifty 5–17 year old Baltimore City children with persistent asthma and a recent exacerbation. Children all had a physician diagnosis of asthma for at least one year and had persistent asthma, either by having a prescription for a controller medication or by having at least 3 days/week of asthma symptoms. All had an asthma exacerbation in the 12 months prior to enrollment. All participants were screened at baseline using a rapid urine cotinine test to exclude smokers. Children with rapid urine cotinine test results that were consistent with second hand smoke exposure were not excluded. At enrollment and every 3 months, subjects underwent disease control characterization by questionnaire and physiologic assessment. As the current cross-sectional study was designed after the onset of MAACS, a convenience sample of study subjects was offered enrollment in the saliva ancillary study at any study visit, leading to enrollment of 58 of the 150 MAACS participants. The adult study was a cross-sectional sample of one hundred twenty 18–55 year old non-smoking physician-diagnosed asthmatics recruited in two clinical settings: 1) the Boston Medical Center (BMC) Allergy and Asthma Clinic at the time of a routine or unscheduled clinical visit and 2) the BMC Emergency Department when presenting for an acute exacerbation. Adults all had a physician diagnosis of asthma, were non-smokers, and were on quick-relief medication for symptom relief. The inclusion criteria were broad to reflect, to the extent possible, the range of asthma that would be seen outside of a research setting. Race and ethnicity for subjects in both studies were self-reported according to guidelines by the United States National Institutes of Health. Non-smoking adult volunteers without asthma who did not have active periodontal disease nor history of major systemic illness were recruited from the Boston University Medical Campus community.

### Ethics Statement

Studies were approved by Institutional Review Boards at Johns Hopkins University (NA-00021358) and Boston University Medical Campus (H-26049). Written informed consent was obtained from parents or guardians on behalf of minors as approved by the Johns Hopkins University Institutional Review Board.

### Study Procedures

#### Clinical Characterization: Pediatric Study

Allergy skin testing was performed at the first study visit to 14 common aeroallergens with controls using the MultiTest device (Lincoln Diagnostics, Decatur, IL). F_E_NO was measured according to American Thoracic Society (ATS) Guidelines using a handheld, FDA-approved analyzer (NIOX TM System, Aerocrine, Sweden) [10]. Spirometry was performed according to American Thoracic Society (ATS) guidelines [11]. A questionnaire that captures medication use [12], health care utilization, and oral health was administered. Days of symptoms over the previous 2 weeks were also captured and a composite variable, maximum symptoms days, was created by taking the maximum number of symptoms days among three of the symptoms variables: nocturnal symptoms, days of slowed activity, and days of exercise-related symptoms [2].

#### Clinical Characterization: Adult Study

A questionnaire covering oral health and history and asthma control, using the Asthma Control Test (ACT) [12] and Asthma Control Questionnaire (ACQ) [13], was administered by the study coordinator. In addition, a brief clinician questionnaire was completed that queried recent control and whether the subject was in exacerbation. Underlying asthma severity using NIH-National Asthma Education and Prevention Program (NAEPP) criteria was assessed for outpatient clinic subjects but not for subjects recruited in the Emergency Department.

#### Oral Exam, Saliva Collection and Processing

The study coordinator carried out a focused oral exam to assess the number of teeth and presence of active tooth and periodontal disease. A masticatory saliva sample was collected using standardized methods as previously described [9]. Briefly, after a 30 minute period of food or liquid abstinence, subjects chew on a neutral substance (Parafilm) and clear their mouth of saliva every 30 seconds into a 60 cc polystyrene tube kept on ice. This was repeated until 6–10 mls of whole saliva are collected. Saliva supernatant was separated by centrifugation at 13,000 g for 20 minutes and stored at −80°C.

#### Nasal Lavage Fluid Collection and Processing

Nasal lavage fluid (NLF) was obtained from study subjects and processed as previously described [14]. NLF samples were considered suitable for further analysis if they had >20,000 non-squamous cells per milliliter.

#### Salivary and Nasal Lavage Analyte Quantification

Supernatants were analyzed on the Qiagen Liquichip apparatus (Luminex) with custom designed 10-plex kits (BioRad) for the following analytes: eotaxin-1/CCL11, RANTES/CCL5, IL-5, IL-6, MIP-1β/CCL4, VEGF, IL-8/CXCL8, IL-1β, MCP-1/CCL2, and IP-10/CXCL10. All saliva supernatants collected were suitable for analysis.

### Statistical Analysis

All analyses were performed with StataSE 12.0 (College Station, TX). The data were explored by graphical display and examination of summary statistics. Salivary inflammatory markers were log_10_-transformed to approximate a normal distribution. Differences between asthmatic and non-asthmatic salivary marker levels were analyzed using the Mann-Whitney U test. Correlation matrices of the salivary inflammatory markers were used to examine relationships among the markers. To capture the overall pattern of salivary inflammation, principal component analyses were performed. Principal component analysis (PCA) is a statistical method that reduces a high dimensional data set, i.e. one that contains many variables, to a few factors that together capture almost all of the information provided by the entire data set. In this study, PCA identified 3 components that captured >75% of the variability observed among all ten analytes, and, as expected with PCA, the components reflected the fact that certain groups of inflammatory markers tended to track together. These factors, or components, reflect the underlying salivary inflammatory pattern and a PC score, which reflects that subject's salivary inflammatory pattern, can be calculated for each component for each subject. For both adult and pediatric data sets, the three principal components were used to calculate principal component scores for each subject. Bivariate analyses were performed to examine relationships between asthma outcomes and potential confounders (oral health and medication variables) and the principal component scores. Covariates that could plausibly be associated with both the salivary inflammatory profile and disease activity were evaluated and included age, sex, BMI, oral health characteristics, corticosteroids, and systemic diseases. Linear and logistic regression were used to model relationships between principal component scores and clinical outcomes. Final models were adjusted for potential confounders identified in the bivariate exploratory analyses. A p value <0.05 was considered statistically significant.

## Results

### Study Population Characteristics

The pediatric subjects recruited for this study were evenly split between male and female, predominantly African American, and from socioeconomically disadvantaged households (Table 1). Their mean age was 12 years. Subjects had persistent asthma by NAEPP EPR-3 criteria and had an exacerbation in the 12 months prior to enrollment. Consistent with other populations of urban asthmatic children, 86% were atopic (Table 2): 85% were sensitized to an indoor allergen. Sixty-nine percent were sensitized to cat, 67% to rat, 59% to cockroach, and 47% to both mouse and dust mite. There were more women (61%) than men in the adult study population. Approximately two thirds were African American and one quarter of Hispanic/Latino ethnicity (Table 1), with a mean age of 43 years. There were 25 adults without asthma; 36% were male with a mean+/−SD age of 36+/−11. Twelve percent were of Hispanic ethnicity and 44% of African-American race with the remainder Caucasian. Further characteristics of the asthmatic study populations are depicted in Tables 2 and 3. Oral health characteristics are depicted in Table S1A and B in the Supporting Information. Six percent of adult asthmatics had active periodontal disease; the mean number of teeth was 28 (interquartile range 23–30; maximum of 32 including third molars).

**Table 1:** Sociodemographic Characteristics.

Characteristic	Adults, n (%)	Children, n (%)
**Age** (y), mean±SD	43.8±14.4	11.7±4.0
**Gender**		
Female	74 (60.7)	30 (51.7)
Male	48 (39.3)	28 (48.3)
**Race**		
Black/African American	81 (66.4)	51 (87.9)
White	34 (27.9)	1 (1.7)
Other/unknown	7 (5.7)	6 (10.4)
**Ethnicity**		
Hispanic/Latino	29 (23.8)	4 (6.9)

**Table 2:** Asthma and Allergic Disease Characteristics, Pediatric Population.

Characteristic	n (%)
Atopic (≥1+SPT)	50 (86.2)
F_E_NO (ppb), median (IQR)*	20 (14–38)
FEV1, % predicted (mean ±SD)§	94±18
FEV1/FVC, % (mean ±SD)§	80±8
Controller medication, current	41 (70.7)
Acute Visit for asthma in previous 3 months¥	17 (30.4)
Short-acting beta-agonist use, days/2 weeks (mean ±SD)¥	3.6±4.5
Eczema, ever	30 (51.7)
Allergic Rhinitis or Hayfever, current	44 (75.9)
Allergen Immunotherapy, ever	2 (3.5)

*n = 57,

n = 55,

n = 56.

F_E_NO: Fractional Exhaled Nitric Oxide, SPT: Skin Prick Test, FEV1: Forced Expiratory Volume in the 1^st^ second, FVC: Forced Vital Capacity.

**Table 3:** Asthma and Allergic Disease Characteristics, Adult Population.

Characteristic	n (%)
**Study Visit Location**	
Asthma Specialist Clinic	89 (73.0)
Emergency Department	33 (27.0)
**Inhaled Corticosteroids, current**	95 (77.9)
**Prednisone, current**	21 (17.2)
**ACQ score, mean±SD**	12.2±9.1
**ACT score, mean±SD**	15.6±5.4
**Asthma Control, Clinician Assessment** *	
Well Controlled	17 (14.2)
Uncontrolled	50 (41.6)
Poorly Controlled	53 (44.2)
**Current Exacerbation, Clinician Assessment** *	48 (40.0)
**Eczema, ever**	28 (23.0)
**Allergic Rhinitis or Hayfever, current**	82 (67.2)
**Allergen Immunotherapy, current**	14 (11.5)

*n = 120.

ACQ: Asthma Control Questionnaire, ACT: Asthma Control Test.

Both populations reflected a range of asthma severity. Although severity per se was not assessed in the pediatric population, their medication regimens indicate a range of severity: of the 41 who were on a controller, 4 were on a leukotriene modifier (LTM) only, 12 were on inhaled corticosteroids (ICS) only, 10 were on ICS plus a LTM, 8 were on ICS plus a long-acting beta-agonist (LABA), and 7 were on ICS plus LABA plus LTM. For the adult population, 75% of outpatients had moderate or severe persistent disease (4.8% were mild intermittent, 20.5% mild persistent, 48.2 moderate persistent, and 26.5% severe persistent); 40% were in exacerbation at enrollment. Ninety-five of the adults (78%) were on a controller medication; 34 were on ICS alone and 61 were on ICS plus LABA.

All subjects in both populations (including children as young as 5 years) who agreed to participate were able to provide an adequate sample of masticatory whole saliva for analysis. Of the adults who provided nasal lavage (n = 122), approximately 65% had nasal lavage that was suitable for analysis (see Methods).

### Characterization of the Asthmatic Salivary Inflammatory Profile

Based on a broad preliminary screening of a 72-analyte panel of inflammatory mediators, we had previously refined a 10-plex suite of analytes that were readily detectable and had robust inter-individual variability among asthmatics [9]. The 10-analyte panel, with lower limit of detection and median salivary concentrations for both populations is depicted in Table 4. A comparison of salivary marker concentrations between adults with and without asthma shows that IL-5, IL-6, MCP-1, and VEGF concentrations were present in statistically significantly higher concentrations in saliva collected from asthmatics compared to controls, and the other markers were not (Figure S1, Supporting Information).

**Table 4:** Salivary Marker Panel and Summary Statistics.

Marker	LLOD	Pediatric Study	Adult Study
		Median (IQR)	Median (IQR)
Eotaxin-1/CCL11	1.0	7.7 (3.7–13.3)	2.3 (<LLOD-5.3)
RANTES/CCL5	0.6	6.7 (4.7–10.4)	2.9 (1.3–5.6)
IL-5	0.2	2.5 (1.9–3.4)	2.9 (1.8–4.3)
IL-6	0.2	5.5 (2.8–12.6)	2.3 (1.3–4.1)
MIP1-β/CCL4	0.3	4.1 (2.9–9.0)	2.1 (1.3–3.7)
VEGF	1.0	327 (176–563)	211 (95–371)
IL-8/CXCL8	0.2	83.9 (44.3–113.0)	83.1 (45.9–140.0)
IL-1β	0.3	13.3 (5.9–33.2)	10.7 (3.2–27.7)
MCP-1/CCL2	0.3	43.8 (20.9–66.9)	38.7 (17.5–62.7)
IP-10/CXCL10	1.0	866 (462–1920)	168 (43–433)

Concentrations in pg/ml.

IL: Interleukin, RANTES: Regulated on Activation Normal T-cell Expressed and Secreted,

MIP: Macrophage Inflammatory Protein, VEGF: Vascular Endothelial Growth Factor,

MCP: Macrophage Chemoattractant Protein, IP-10: Interferon gamma inducible Protein 10 kd.

**Figure 1:**
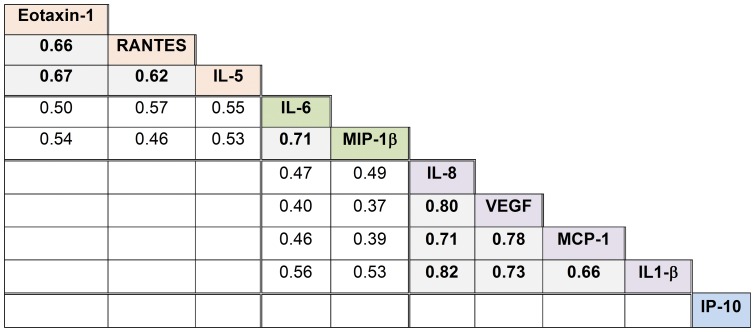
Correlation matrix for 10-analyte salivary inflammatory marker panel. The mean of the pediatric and adult populations' Pearson's correlation coefficients for each pairwise correlation of log_10_ transformed salivary markers is displayed. Correlation coefficients are displayed if p<0.01 for both pediatric and adult populations for the pairwise relationship. **Bolded** correlation coefficients are >0.6.

We found the strongest correlations amongst saliva mediators that reflect similar immunologic/inflammatory pathways (Figure 1). Specifically, in both the pediatric and adult populations, there were strong, statistically significant correlations amongst the eosinophil-related markers eotaxin-1/CCL11, RANTES/CCL5, and IL-5 (r = 0.62–0.67). Similarly, there were strong correlations amongst myeloid markers of innate immune activation including IL-1β, MCP-1/CCL2, and IL-8/CXCL8 (r = 0.66–0.82). Because the analysis included multiple comparisons, we only depict correlations where p<0.01 in *both* populations in Figure 1. Full correlation matrices for the pediatric and adult populations can be found in the Supporting Information (Figures S2 and S3).

While vascular endothelial growth factor (VEGF) is not considered a marker of innate immune responses, we found strong correlations within samples amongst VEGF and the myeloid markers of innate immune activation (IL-8/CXCL8, MCP-1/CCL2, and IL-1β) in both populations (r = 0.73–0.79). IL-6 and MIP1β emerged as general markers of inflammation as these markers had low to moderate correlations with most of the other 8 inflammatory markers (r = 0.36–0.56). Of note, IP-10/CXCL10 (abundant in all samples) had no strong correlation with any other markers.

### Principal Component Analysis of Salivary Inflammatory Mediators in Adults and Children

**Figure 2:**
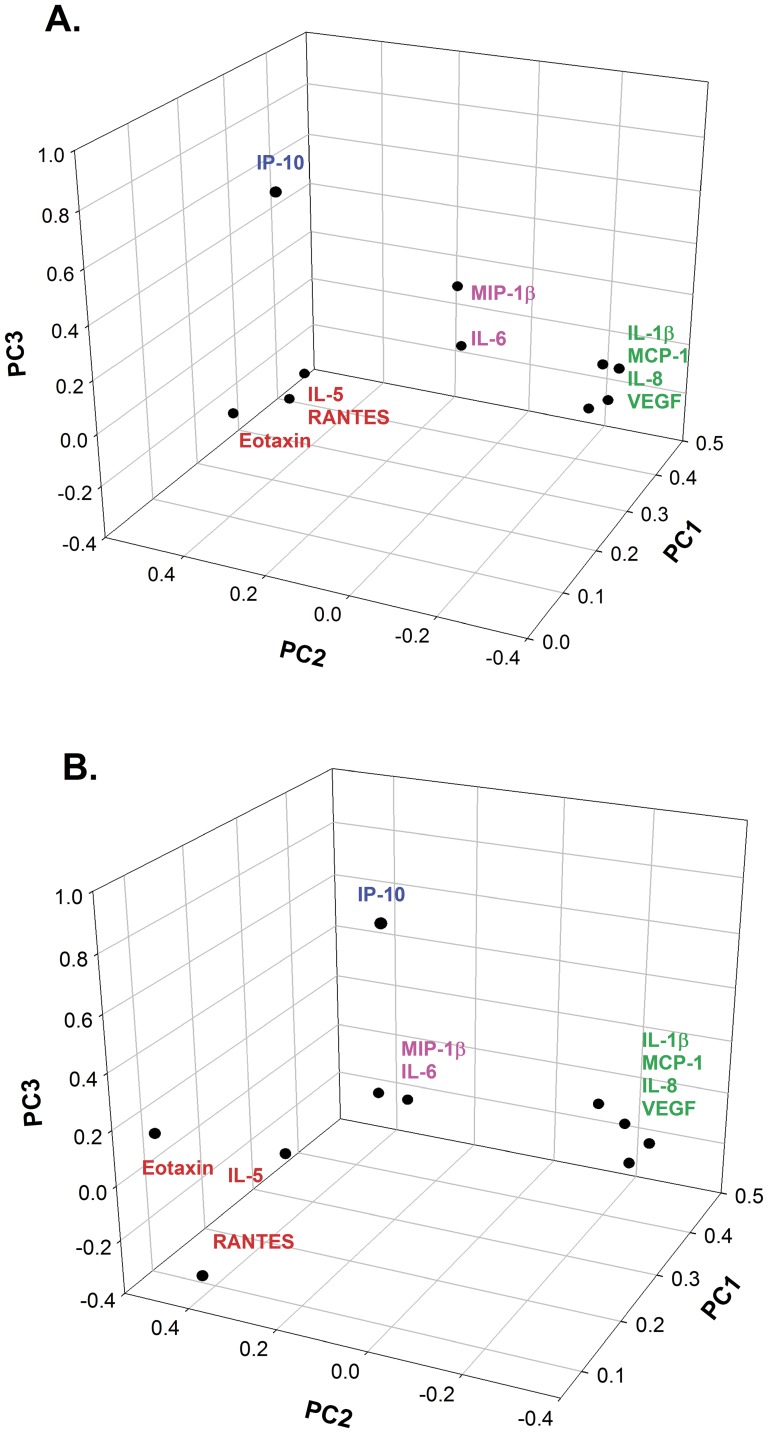
3-D plots of component loadings for the pediatric (A) and adult (B) populations.

Because we were most interested in understanding if the pattern of salivary inflammatory markers reflected clinical markers of disease, we carried out principal component analysis (PCA) of mediator concentrations in both study populations. The first three principal components accounted for 85% and 74% of the variability in the pediatric and adult population, respectively (see Table S2 in the Supporting Information). PCA performed independently in both the pediatric and adult populations identified strikingly similar principal components. For both populations, each analyte's coefficient for PC1 was positive for all ten markers, so that this component reflects the general degree of inflammation across all markers, with slightly greater weight given to the myeloid/innate-immunity related markers as these latter markers have larger coefficients for PC1 than the other markers. The second component reflects the difference between the eosinophil-related markers and the myeloid/innate immunity-related markers (Table 5). Specifically, a higher component 2 score for an individual subject indicates higher concentrations of eosinophil-related markers relative to concentrations of myeloid/innate immune markers while a lower, and a negative component 2 score indicates higher concentrations of myeloid/innate immune markers relative to eosinophil-related markers. PC2 reflects the relative contributions of these markers in this way because the eosinophil-related markers have positive coefficients and the myeloid/innate markers have negative coefficients for PC2. Therefore, a PC2 score around 0 reflects similar concentrations of both eosinophil- and innate immune-related markers. The third component generally reflects IP-10/CXCL10 concentrations as each analyte's coefficient is close to 0 for all markers except for IP-10/CXCL10. Operationally, to calculate a PC1 scores for a given individual, the concentration of each of the individual's analytes is multiplied by its PC1 coefficient and then each of these is summed and the result is that individual's PC1 score. The same operation is used to calculate an individual's PC2 and PC3 scores. Depicted graphically, the contributions of the individual markers to each of the three PCs in both populations were strikingly similar to each other (Figure 2).

**Table 5:** Contribution of Salivary Markers to Each Principal Component Score.

Marker	PC1		PC2		PC3	
	Adults	Children	Adults	Children	Adults	Children
**Eotaxin/CCL11**	0.11	0.19	0.54	0.48	0.10	−0.14
**RANTES/CCL5**	0.09	0.30	0.42	0.42	−0.35	−0.11
**IL-5**	0.22	0.27	0.37	0.43	−0.07	−0.18
**IL-6**	0.38	0.39	0.25	0.09	0.01	−0.02
**MIP-1β/CCL4**	0.36	0.38	0.30	0.09	−0.02	0.24
**VEGF**	0.39	0.33	−0.31	−0.35	−0.06	0.11
**IL-8/CXCL8**	0.42	0.37	−0.23	−0.30	−0.05	−0.08
**IL-1β**	0.43	0.36	−0.16	−0.28	−0.02	0.06
**MCP-1/CCL2**	0.36	0.35	−0.28	−0.26	−0.11	−0.11
**IP-10/CXCL10**	0.14	0.04	0.06	0.19	0.92	0.92

Principal Component 1 reflects relative similar representation of all markers with slightly greater weight to innate/myeloid markers.

Principal Component 2 reflects greater representation of eosinophil/lymphoid markers relative to innate markers.

Principal Component 3 predominantly reflects IP-10/CXCL10.

IL: Interleukin, RANTES: Regulated on Activation Normal T-cell Expressed and Secreted,

MIP: Macrophage Inflammatory Protein, VEGF: Vascular Endothelial Growth Factor,

MCP: Macrophage Chemoattractant Protein, IP-10: Interferon gamma inducible Protein 10 kd.

### Principal Component Scores and Relationship to Clinical Markers of Disease and Outcomes

For the pediatric population, the PC1 score was associated with nocturnal and exercise-related symptoms, but none of the other symptoms outcomes. The PC2 score was inversely associated with most symptoms outcomes, including maximum symptoms days; cough, wheeze, and chest tightness; nocturnal symptoms, and slowed activity. The PC3 score was positively associated with all symptoms outcomes. These relationships were generally unaffected by adjustment for controller medication use including inhaled steroids, frequency of dental checkups, and report of bleeding gums with brushing (Table 6). No pediatric subjects had used systemic steroids in the 2 weeks prior to study visits. Oral health information is depicted in Table S1A of the Supporting Information. None of the PC scores were independently associated with lung function or F_E_NO, nor were our findings changed when models were adjusted for age and gender (data not shown).

**Table 6:** Relationships between salivary inflammatory marker principal components and measures of asthma control among children with asthma*.

	PC1 Score OR (95% CI)		PC2 Score OR (95% CI)		PC3 Score OR (95% CI)	
	Crude	Adjusted¥	Crude	Adjusted¥	Crude	Adjusted¥
**Max Symptoms Days**	1.01 (0.94–1.10)	1.07 (0.97–1.17)	0.91 (0.81–1.01)	0.90 (0.79–1.02)	**1.31 (1.10–1.56)**	**1.54 (1.23–1.93)**
**Cough, Wheeze, Chest Tightness**	1.04 (0.95–1.13)	1.07 (0.97–1.18)	**0.82 (0.73–0.93)**	**0.79 (0.69–0.91)**	**1.24 (1.04–1.48)**	**1.31 (1.05–1.63)**
**Nocturnal Symptoms**	**1.18 (1.04–1.34)**	**1.20 (1.02–1.40)**	**0.76 (0.64–0.90)**	**0.73 (0.59–0.90)**	**1.28 (1.01–1.61)**	**1.39 (1.03–1.89)**
**Exercise-related Symptoms**	**1.10 (1.00–1.22)**	**1.12 (1.00–1.26)**	0.89 (0.78–1.01)	0.91 (0.80–1.05)	**1.37 (1.11–1.68)**	**1.50 (1.15–1.95)**
**Slowed Activity**	0.97 (0.88–1.07)	0.98 (0.88–1.10)	0.88 (0.77–1.01)	**0.79 (0.66–0.95)**	**1.54 (1.25–1.90)**	**2.28 (1.69–3.06)**

*regression models include all three PC scores; relationships were modeled using logistic regression;

**Bolded results** are statistically significant at p<0.05; PC = principal component; crude models: n = 56.

adjusted for controller medication, frequency of dental checkups, and report of gums bleeding with tooth brushing, n = 49.

Principal Component 1 reflects relative similar representation of all markers with slightly greater weight to innate/myeloid markers.

Principal Component 2 reflects greater representation of eosinophil/lymphoid markers relative to innate markers.

Principal Component 3 predominantly reflects IP-10/CXCL10.

**Figure 3:**
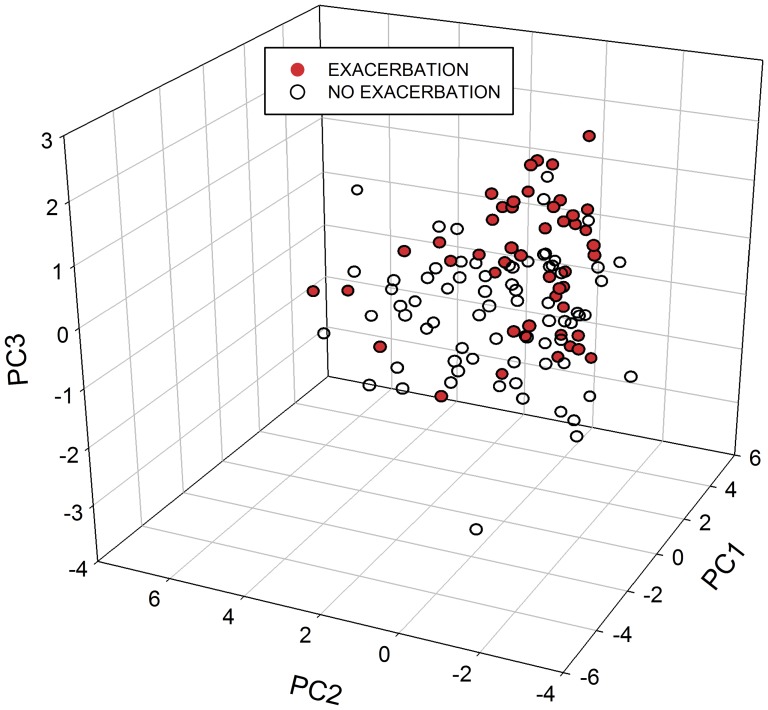
Three-dimensional display of subject principal component scores with respect to clinician-determined exacerbation in adults, n = 120.

For the adult population, PC1 and PC3 scores were associated with acute loss of asthma control, as captured by ACQ score and current exacerbation, while the PC2 score was not associated with any measures of asthma control (Table 7). Consistent with these findings, clinician-assessed asthma control was associated with PC1 and PC3 scores (p = 0.05 and 0.006, respectively). Notably, neither PC1 nor PC3 was associated with ACT score, which reflects control over the previous 4 weeks, rather than the 1 week time period captured by ACQ score. The associations were unchanged in models adjusted for concomitant inhaled and systemic steroid use, or by key oral health characteristics that could contribute to oral inflammation. Details of adult oral health are provided in Table S1B in the Supporting Information. The associations were also unchanged when models were adjusted for age and gender. When individual subject scores for each principal component were displayed, there was a segregation of patients by the categorical variable of clinician-determined exacerbation (Figure 3).

**Table 7:** Relationships between salivary inflammatory marker principal components and measures of asthma control among adults with asthma*.

	PC1 Score β/OR (95% CI)		PC2 Score β/OR (95% CI)		PC3 Score β/OR (95% CI)	
	Crude	Adjusted¥	Crude	Adjusted¥	Crude	Adjusted¥
**ACT Score**	−0.22 (−0.72–0.29)	−0.22 (−0.72–0.28)	−0.19 (−0.80–0.42)	−0.26 (−0.88–0.35)	−0.19 (−1.17–0.79)	−0.52 (−1.56–0.53)
**ACQ Score**	**0.98 (0.15–1.80)**	**0.96 (0.12–1.80)**	0.09 (−0.91–1.09)	0.27 (−0.76–1.29)	1.04 (−0.56–2.65)	1.29 (−0.46–3.03)
**Current Exacerbation**	**1.24 (1.01–1.54)**	**1.32 (1.04–1.68)**	0.87(0.67–1.13)	0.91 (0.69–1.20)	**2.30 (1.46–3.62)**	**2.41 (1.41–4.10)**

*regression models include all three PC scores; relationships were modeled using multiple linear or logistic regression; **bolded results** are statistically significant at p<0.05; PC = principal component.

adjusted for number of teeth, gingivitis, oral prednisone, inhaled corticosteroids.

Principal Component 1 reflects relative similar representation of all markers with slightly greater weight to innate/myeloid markers.

Principal Component 2 reflects greater representation of eosinophil/lymphoid markers relative to innate markers.

Principal Component 3 predominantly reflects IP-10/CXCL10.

ACQ: Asthma Control Questionnaire, ACT: Asthma Control Test.

We explored whether systemic conditions such as obesity, hypertension, gastroesophageal reflux disease, diabetes, and autoimmune disease, could have affected the associations we found between salivary marker PC scores and asthma control. Table S3 in the Supporting Information depicts the prevalence of systemic comorbidities in our adult population. When we adjusted the final models for comorbidities, there was no change in the significant associations between PC scores and markers of asthma control (Table S4 in the Supporting Information). Our pediatric population did not have any significant comorbidities, and consistent with our results in adults, there was no effect of body mass index on our findings.

### Relationship Between Salivary and Nasal Lavage Fluid Analytes

Eighty adult subjects provided NL specimens that were adequate for marker analysis. There was no difference between these and the 42 whose NL samples were inadequate with respect to age, gender, race, or ethnicity (Table S5, Supporting Information). To address the question of whether the inflammatory composition of the upper airways is reflected in the oral cavity, we compared concurrently collected NLF and saliva marker levels. There were no significant correlations between NL and saliva levels of any of the 10 markers, suggesting that the nasal and oral compartments are not mutually reflective with respect to the inflammatory markers measured (Table S6, Supporting Information). In addition, we did not find any association between NL markers and asthma control (not shown).

## Discussion and Conclusions

This is the first study, to our knowledge, to demonstrate that the pattern of salivary inflammatory markers may serve as a means for assessing asthma control. Specifically, we found patterns of inflammatory markers within saliva samples that were biologically consistent in both pediatric and adult asthmatics. The observation that markers that are eosinophil-related and those that are myeloid/innate-related were strongly correlated with one another supports the notion that biologically valid measurements of inflammation can be made in saliva. Furthermore, the patterns of inflammatory markers identified by PCA were strikingly similar in the two populations, supporting the notion that the range of patterns of salivary inflammation across all asthmatics may be described using the same principal components. Perhaps more importantly, the patterns of salivary markers were also associated with measures of current or very recent asthma control, independent of oral health and corticosteroid use. Not only were the inflammatory patterns within saliva and across people similar in both populations, but there were also some similarities between the populations with respect to the types of inflammatory patterns associated with uncontrolled disease. Our findings suggest that saliva has the potential to be a non-pulmonary, readily available biospecimen for correlating with clinical markers of asthma control, and may be useful in the clinical setting with the development of point-of-care devices [9].

The field of saliva diagnostics for diseases outside of the oral cavity is young [15]. Several small studies have examined the relationship between individual salivary markers and asthma disease status [16], [17]. In contrast, we sought to determine the utility of examining a multi-marker suite of relevant markers in asthmatics to identify salivary profiles that relate to asthma control. By performing multiplex analysis in two entirely independent populations of asthmatics covering a range of underlying severity and control, we have shown that salivary inflammatory marker analysis can provide discrete insights on current asthma control and recent symptoms.

A notable finding in this study is the tight correlation amongst certain groups of inflammatory markers within samples, many of which were biologically related. Other investigators applying multianalyte testing on other biospecimens have found correlations amongst biologically related mediators, but using a different panel of analytes [18]. The salivary markers with the strongest correlations within samples fit generally into 2 groups: markers of eosinophil/lymphocyte activation and markers of myeloid/innate immune activation. IL-6 and MIP-1β each correlated significantly with markers from both other groups, albeit not as strongly as with each other. IL-6 and MIP1β are pleiotropic inflammatory molecules with multiple cell sources and sites of action, and from a biologic perspective, do not fall readily into either of the other groups with strong intragroup correlations within samples. While VEGF is not currently defined as a marker of innate immune activation, it has been identified as being contributory to pathogenesis of airway and vascular changes in asthma [19], [20] through mechanisms which may have direct interplay with myeloid activation [21]. It is notable that IP-10/CXCL10, readily measurable in virtually all saliva samples as it is in varied other biospecimens [22]–[26], figured prominently as a marker that is reflective of disease control in our principal component analysis (PC3), yet did not correlate with other markers in either population.

We also found that the salivary inflammatory profile was strongly associated with measures of uncontrolled asthma, and the pediatric and adult population shared some specific patterns that were linked to loss of control. PC3, reflective of salivary IP-10/CXCL10 concentrations primarily, was positively associated with exacerbation in adults and a range of asthma symptoms in children. PC1, reflective of elevated inflammatory markers overall, but with greater weight to the myeloid/innate markers, was associated with uncontrolled asthma in adults and nocturnal and exercise-related symptoms in children. The lack of association between PC scores and ACT may be due to the fact that ACT captures control over the preceding 4 weeks, while ACQ in adults and the asthma symptom measures in children capture control over the preceding 1 and 2 weeks, respectively. Not all of our findings were similar in both populations, and although the reasons for this are unclear, it is possible that differences between these two populations, such as age, socioeconomic status, environmental exposures, are responsible for results that were not consistent between the two groups. In adults, there were no associations between PC2 and markers of control, while PC2 score was inversely associated with composite symptoms in children. This is likely due to differences in underlying phenotypes of pediatric and adult asthma.

As reported recently, in a predominantly white Swedish population of adults ranging from 20–89 years, certain salivary markers have been found to be associated with systemic conditions, including diabetes, hypertension, and joint diseases [6]. Our findings were not affected when we adjusted for these and other systemic conditions in our models. This could be explained by the fact that our study was specifically recruiting individuals with a primary disorder, where any changes from systemic disease were too modest compared to those from asthma. In addition, the mediator panel in the Rathnayake study differed from ours, as was their statistical analysis. While we did not see a confounding effect of oral health on the associations between salivary profiles and asthma control in our study populations, others have found associations between certain inflammatory markers and oral health characteristics, including pocket depth, bleeding on probing [27], and periodontal disease status [28]–[30]. Further studies are warranted in asthmatics with a range of periodontal/gingival disease to definitively conclude that oral health does not confound associations between salivary marker profiles and asthma control.

Our comparison of markers in nasal lavage fluid and saliva suggest that these two compartments are not reflective of each other, contrary to our premise that the oral cavity would be reflective of the inflammatory composition of the upper airways. Because we did not assess lower airways inflammation in this study, future studies should examine associations between oral and lower airways inflammatory patterns. Even if the salivary inflammatory profile does not reflect the inflammatory profile of either the upper or lower airways, our findings still suggest that saliva can serve as a ‘standalone’ source of useful asthma biomarkers. Furthermore, our finding that nasal lavage mediators did not correlate with markers of disease control is consistent with those of other investigators [31], [32].

Determining the cellular source of the markers measured, while relevant to understanding the mechanisms by which certain markers are related to asthma disease activity, was beyond the scope of the current study. In fact, there are very few studies on the cellular source(s) in the mouth of the markers we studied, and none in asthma [33]–[35]. However, these findings do highlight the potential of salivary inflammatory profiling to lend insight into the pathogenesis of asthma. For example, IP-10/CXCL10, reflected by principal component 3, was strongly associated with uncontrolled asthma in both the pediatric and adult populations. The fact that principal component 2 (reflective of greater eosinophilic inflammation relative to myeloid/innate inflammation) was either not associated, or inversely associated, with uncontrolled asthma, while principal components 1 and 3 were positively associated with uncontrolled asthma, supports the notion that triggers of the innate immune response may be more responsible for acute deteriorations in asthma than triggers of eosinophilic inflammation.

Although the salivary inflammatory profile is strongly linked to asthma control in our two independent study populations, whether this is the case because it reflects the inflammatory composition of the airways, systemic circulation, or only the oral cavity is not clear. Our findings do not suggest that the salivary inflammatory profile is a reflection of upper airway inflammation as assessed by nasal lavage. Salivary inflammatory markers are also not likely a reflection of systemic inflammation as the majority of oral fluid is from the exocrine secretion by salivary glands, and several groups have demonstrated that the oral compartment is not reflective of serum [36], [37]. Taken together, these data suggest that our salivary inflammatory profile is a faithful representation of the oral compartment but not representative of the upper airway or systemic circulation. Although our study was not designed to determine the mechanism by which inflammatory markers in the oral cavity are related to asthma control, the study's findings support the conduct of studies to elucidate the mechanism, including studies that directly compare lower airways and salivary inflammation. In addition, it is possible that environmental exposures influence the inflammatory profile of the oral cavity and this hypothesis should also be tested in future studies.

There are several limitations to our study. First, the pediatric and adult studies were not matched with respect to physiologic or clinical assessments. While this limited our ability to compare salivary marker patterns with physiologic parameters in the adults as we could in children, there were meaningful clinical parameters that were associated with marker profiling in both populations. Second, while our results permit drawing conclusions regarding asthmatics and disease control based on salivary profiling as a population, our findings do not permit making predictions about individual patients. Our cross-sectional study findings support the conduct of longitudinal studies to determine how salivary profiles behave over time and to potentially predict future exacerbations. If so, salivary inflammatory profiling could be incorporated into treatment algorithms with the objective of reducing the risk of an exacerbation. Third, we did not compare our results in saliva to those from an established lower respiratory source of asthma markers, such as bronchoalveolar lavage fluid or induced sputum. However, our results and others' [7], [8] suggest that non-respiratory specimens can be an informative source of disease markers.

In conclusion, we provide evidence for using multianalyte profiling of particular salivary inflammatory markers in assessing disease control in asthma. Specifically, the salivary inflammatory profile was associated with measures of current or very recent asthma control, with a general increase in concentrations of the ten inflammatory markers and an increase in IP-10 being associated with measures of uncontrolled asthma in both children and adults. Further development of salivary inflammatory profiling for clinical application depends upon demonstrating that the salivary inflammatory profile predicts future loss of control and identifying clinical decision-making thresholds that both predict loss of control and will prevent exacerbations.

## Supporting Information

Figure S1 **Boxplots of salivary inflammatory marker concentrations among adults with and without asthma: (a) Eotaxin-1, (b) IL-1β, (c) IL-5, (d) IL-6, (e) IL-8, (f) IP-10, (g) MCP-1, (h) MIP-1β, (i) RANTES, (j) VEGF.** Mann-Whitney U tests were used to compare differences between asthmatics (left boxplot for each marker) and controls (right boxplot for each marker).
(DOCX)

Figure S2 **Correlation matrix (Pearson's r) of salivary markers in pediatric asthmatics, n = 58.**
(DOCX)

Figure S3 **Correlation matrix (Pearson's r) of salivary markers in adult asthmatics, n = 122.**
(DOCX)

Table S1 **Oral health characteristics.**
(DOCX)

Table S2 **Principal Components for panel of 10 inflammatory markers in saliva of asthmatics.**
(DOCX)

Table S3 **Comorbid conditions, adult population (n-118).**
(DOCX)

Table S4 **Effect of systemic diseases on relationship between salivary marker PC scores and asthma disease control in adults.**
(DOCX)

Table S5 **Comparison of demographic characteristics of subjects who had nasal lavage fluid adequate for analysis with entire adult population.**
(DOCX)

Table S6 **Correlations for nasal lavage vs. salivary inflammatory markers concentrations.**
(DOCX)
