# Supplementary material for: Salivary Inflammatory Mediator Profiling and Correlation to Clinical Disease Markers in Asthma
Source: PLoS One. 2014 Jan 7;9(1):e84449. doi: 10.1371/journal.pone.0084449 (PMC3883659; doi:10.1371/journal.pone.0084449)
Supplement: Figure S1 — Boxplots of salivary inflammatory marker concentrations among adults with and without asthma: (a) Eotaxin-1, (b) IL-1β, (c) IL-5, (d) IL-6, (e) IL-8, (f) IP-10, (g) MCP-1, (h) MIP-1β, (i) RANTES, (j) VEGF. Mann-Whitney U tests were used to compare differences between asthmatics (left boxplot for each marker) and controls (right boxplot for each marker). (DOCX) [file pone.0084449.s001.docx]

**FIGURE S1**. Boxplots of salivary inflammatory marker concentrations among adults with and without asthma: (a) Eotaxin-1, (b) IL-1β, (c) IL-5, (d) IL-6, (e) IL-8, (f) IP-10, (g) MCP-1, (h) MIP-1β, (i) RANTES, (j) VEGF. Mann-Whitney U tests were used to compare differences between asthmatics (left boxplot for each marker) and controls (right boxplot for each marker).

**FIGURE S1**. (continued). Boxplots of salivary inflammatory marker concentrations among adults with and without asthma
