# Supplementary material for: Salivary Inflammatory Mediator Profiling and Correlation to Clinical Disease Markers in Asthma
Source: PLoS One. 2014 Jan 7;9(1):e84449. doi: 10.1371/journal.pone.0084449 (PMC3883659; doi:10.1371/journal.pone.0084449)
Supplement: Figure S2 — Correlation matrix (Pearson's r) of salivary markers in pediatric asthmatics, n = 58. (DOCX) [file pone.0084449.s002.docx]

**FIGURE S2**. Correlation matrix (Pearson’s r) of salivary markers in pediatric asthmatics, n=58.

| **Eotaxin-1** |  |  |  |  |  |  |  |  |  |
| --- | --- | --- | --- | --- | --- | --- | --- | --- | --- |
| **0.80** | **RANTES** |  |  |  |  |  |  |  |  |
| **0.73** | **0.92** | **IL-5** |  |  |  |  |  |  |  |
| **0.38** | **0.65** | **0.60** | **IL-6** |  |  |  |  |  |  |
| **0.45** | **0.56** | **0.50** | **0.73** | **MIP-1β** |  |  |  |  |  |
| 0.04 | 0.20 | 0.12 | **0.54** | **0.58** | **IL-8** |  |  |  |  |
| -0.16 | 0.12 | 0.06 | **0.48** | **0.43** | **0.83** | **VEGF** |  |  |  |
| 0.001 | 0.25 | 0.21 | **0.55** | **0.50** | **0.79** | **0.80** | **MCP-1** |  |  |
| -0.06 | 0.19 | 0.13 | **0.61** | **0.62** | **0.84** | **0.77** | **0.69** | **IL1-β** |  |
| 0.15 | 0.18 | 0.12 | 0.07 | 0.27 | -0.15 | 0.02 | -0.12 | -0.04 | **IP-10** |

p<0.01 **bolded**
