# Supplementary material for: Salivary Inflammatory Mediator Profiling and Correlation to Clinical Disease Markers in Asthma
Source: PLoS One. 2014 Jan 7;9(1):e84449. doi: 10.1371/journal.pone.0084449 (PMC3883659; doi:10.1371/journal.pone.0084449)
Supplement: Figure S3 — Correlation matrix (Pearson's r) of salivary markers in adult asthmatics, n = 122. (DOCX) [file pone.0084449.s003.docx]

**FIGURE S3**. Correlation matrix (Pearson’s r) of salivary markers in adult asthmatics, n=122.

| **Eotaxin-1** |  |  |  |  |  |  |  |  |  |
| --- | --- | --- | --- | --- | --- | --- | --- | --- | --- |
| **0.50** | **RANTES** |  |  |  |  |  |  |  |  |
| **0.58** | **0.29** | **IL-5** |  |  |  |  |  |  |  |
| **0.45** | **0.40** | **0.40** | **IL-6** |  |  |  |  |  |  |
| **0.52** | **0.34** | **0.52** | **0.66** | **MIP-1β** |  |  |  |  |  |
| -0.16 | -0.03 | 0.12 | **0.41** | **0.37** | **IL-8** |  |  |  |  |
| **-0.27** | -0.19 | 0.14 | **0.33** | **0.28** | **0.74** | **VEGF** |  |  |  |
| -0.19 | -0.10 | 0.01 | **0.34** | **0.25** | **0.64** | **0.75** | **MCP-1** |  |  |
| 0.01 | -0.04 | 0.17 | **0.50** | **0.41** | **0.78** | **0.68** | **0.62** | **IL1-β** |  |
| 0.19 | -0.07 | 0.07 | **0.24** | 0.18 | 0.16 | 0.11 | 0.08 | 0.17 | **IP-10** |

p< 0.01 **bolded**
