# Supplementary material for: Salivary Inflammatory Mediator Profiling and Correlation to Clinical Disease Markers in Asthma
Source: PLoS One. 2014 Jan 7;9(1):e84449. doi: 10.1371/journal.pone.0084449 (PMC3883659; doi:10.1371/journal.pone.0084449)
Supplement: Table S1 — Oral health characteristics. (DOCX) [file pone.0084449.s004.docx]

**TABLE S1A. ORAL HEALTH CHARACTERISTICS**

| **Characteristic** | **Adults** | **Children^§^** |
| --- | --- | --- |
| Usual interval between regular dentist visits (months), median (IQR) | 9.5 (6-12) | 6 (6-12)* |
| Frequency of tooth brushing, n (%) | | |
| < 1/day | 6 (4.9) | 0 (0) |
| 1/day | 15 (12.3) | 22 (38.6) |
| 2+/day | 101 (82.8) | 35 (61.4) |
| Blood after brushing, n (%) | 35 (28.7) | 17 (29.8) |

**^§^**n=57, *n=51

**TABLE S1B. ADDITIONAL ORAL HEALTH CHARACTERISTICS**

|  | Adults (n=122) |
| --- | --- |
| Characteristic | n (%) |
| **Dental History** | |
| Painful gums | 16 (13.1) |
| Pain with chewing | 14 (11.5) |
| **Oral Exam Findings** | |
| Clinician Assessment of Dental Status |  |
| No Teeth | 3 (2.5) |
| Poor | 15 (12.5) |
| Good | 69 (57.5) |
| Not noted | 33 (27.5) |
| Number of teeth, median (IQR) | 28 (23-30) |
| Plaque deposits | 41 (33.6) |
| Cavities | 18 (14.8) |
| Gingivitis | 6 (4.9) |
