# Supplementary material for: Salivary Inflammatory Mediator Profiling and Correlation to Clinical Disease Markers in Asthma
Source: PLoS One. 2014 Jan 7;9(1):e84449. doi: 10.1371/journal.pone.0084449 (PMC3883659; doi:10.1371/journal.pone.0084449)
Supplement: Table S2 — Principal Components for panel of 10 inflammatory markers in saliva of asthmatics. (DOCX) [file pone.0084449.s005.docx]

**TABLE S2. PRINCIPAL COMPONENTS FOR PANEL OF 10 INFLAMMATORY MARKERS IN SALIVA OF ASTHMATICS**

|  | **% of variability in all ten inflammatory markers explained** | | **Cumulative % variability explained** | |
| --- | --- | --- | --- | --- |
|  | **Adults** | **Children** | **Adults** | **Children** |
| **PC1** | 38% | 48% | 38% | 48% |
| **PC2** | 26% | 27% | 64% | 75% |
| **PC3** | 10% | 10% | 74% | 85% |
