# Supplementary material for: Salivary Inflammatory Mediator Profiling and Correlation to Clinical Disease Markers in Asthma
Source: PLoS One. 2014 Jan 7;9(1):e84449. doi: 10.1371/journal.pone.0084449 (PMC3883659; doi:10.1371/journal.pone.0084449)
Supplement: Table S3 — Comorbid conditions, adult population (n-118). (DOCX) [file pone.0084449.s006.docx]

**TABLE S3. Comorbid Conditions, Adult Population (n=118)**

| **Characteristic** | **Percent** |
| --- | --- |
| BMI*, median (IQR) | 32 (27-39) |
| Obesity (BMI≥30) | 65% |
| Hypertension | 27% |
| Gastro-esophageal reflux disease | 25% |
| Diabetes | 14% |
| Autoimmune or rheumatologic condition | 5% |
| Coronary Artery Disease | 3% |
| Other GI disease | 2% |
| Cancer | 2% |
| Congestive Heart Failure | 1% |

**n=113*
