# Supplementary material for: Salivary Inflammatory Mediator Profiling and Correlation to Clinical Disease Markers in Asthma
Source: PLoS One. 2014 Jan 7;9(1):e84449. doi: 10.1371/journal.pone.0084449 (PMC3883659; doi:10.1371/journal.pone.0084449)
Supplement: Table S4 — Effect of systemic diseases on relationship between salivary marker PC scores and asthma disease control in adults. (DOCX) [file pone.0084449.s007.docx]

**TABLE S4. Effect of systemic diseases on relationship between salivary marker PC scores and asthma disease control in adults**

|  | Full model*  (n=120) | Full model+BMI  (n=109) | Full model+  Hypertension | Full model+GERD | Full model+diabetes | Full model +autoimmune disease |
| --- | --- | --- | --- | --- | --- | --- |
| **ACT Score**  Beta coefficient (95% CI) |  | |  | | n=116 | |
| PC1 | -0.22  (-0.72-0.28) | -0.29  (-0.72-0.33) | -0.24  (-0.75-0.27) | -0.21  (-0.73-0.31) | -0.22  (-0.73-0.30) | -0.22  (-0.74-0.29) |
| PC2 | -0.26  (-0.88-0.35) | -0.24  (-0.88-0.40) | -0.17  (-0.79-0.45) | -0.25  (-0.88-0.37) | -0.21  (-0.84-0.42) | -0.25  (-0.88-0.37) |
| PC3 | -0.52  (-1.56-0.53) | -0.69  (-1.88-0.49) | -0.52  (-1.58-0.54) | -0.65  (-1.72-0.42) | -0.59  (-1.65-0.49) | -0.60  (-1.67-0.47) |
| **ACQ Score**  Beta coefficient (95% CI) |  |  |  |  |  |  |
| PC1 | **0.96**  **(0.12-1.80)** | **0.86**  **(-0.01-1.73)** | **1.05**  **(0.20-1.90)** | **1.01**  **(0.13-1.89)** | **1.00**  **(0.14-1.87)** | **1.05**  **(0.18-1.92)** |
| PC2 | 0.27  (-0.76-1.29) | 0.36  (-0.69-1.42) | 0.08  (-0.96-1.12) | 0.27  (-0.77-1.32) | 0.18  (-0.88-1.24) | 0.28  (-0.76-1.32) |
| PC3 | 1.29  (-0.46-3.03) | 0.76  (-1.19-2.72) | 1.01  (-0.76-2.79) | 1.34  (-0.47-3.14) | 1.16  (-0.65-2.96) | 1.14  (-0.65-2.94) |
| **Exacerbation**  OR (95% CI) |  |  |  |  |  |  |
| PC1 | **1.32**  **(1.04-1.68)** | **1.31**  **(1.03-1.66)** | **1.34**  **(1.06-1.71)** | **1.32**  **(1.03-1.68)** | **1.34**  **(1.05-1.70)** | **1.37**  **(1.07-1.75)** |
| PC2 | 0.91  (0.69-1.20) | 0.92  (0.70-1.21) | 0.89  (0.67-1.18) | 0.90  (0.69-1.19) | 0.90  (0.68-1.19) | 0.90  (0.69-1.19) |
| PC3 | **2.41**  **(1.41-4.10)** | **2.04**  **(1.17-3.56)** | **2.28**  **(1.34-3.90)** | **2.30**  **(1.35-3.92)** | **2.33**  **(1.37-4.00)** | **2.29**  **(1.35-3.91)** |

*Full model is adjusted for number of teeth, gingivitis, inhaled corticosteroids, and oral corticosteroids

p≤0.05 indicated in **bold**.
