# Supplementary material for: Salivary Inflammatory Mediator Profiling and Correlation to Clinical Disease Markers in Asthma
Source: PLoS One. 2014 Jan 7;9(1):e84449. doi: 10.1371/journal.pone.0084449 (PMC3883659; doi:10.1371/journal.pone.0084449)
Supplement: Table S5 — Comparison of demographic characteristics of subjects who had nasal lavage fluid adequate for analysis with entire adult population. (DOCX) [file pone.0084449.s008.docx]

**TABLE S5. Comparison of DEMOGRAPHIC characteristics OF subjects who had nasal lavage fluid adequate for analysis with the entire adult population**

|  | **Saliva Population** (n=122) | **Nasal lavage subpopulation** (n=80) |
| --- | --- | --- |
| **Age (y), median (IQR)** | 43 (31-55) | 42 (32-54) |
| **Sex, female** | 74 (61%) | 49 (61%) |
| **Hispanic ethnicity** | 29 (24%) | 19 (24%) |
| **Race** | | |
| Black | 81 (66%) | 60 (75%) |
| White | 34 (28%) | 17 (21%) |
