# Supplementary material for: Salivary Inflammatory Mediator Profiling and Correlation to Clinical Disease Markers in Asthma
Source: PLoS One. 2014 Jan 7;9(1):e84449. doi: 10.1371/journal.pone.0084449 (PMC3883659; doi:10.1371/journal.pone.0084449)
Supplement: Table S6 — Correlations for nasal lavage vs. salivary inflammatory markers concentrations. (DOCX) [file pone.0084449.s009.docx]

**Table S6. Correlations* for nasal lavage vs. salivary inflammatory marker concentrations ***

|  | Pearson correlation coefficient | p-value |
| --- | --- | --- |
| Eotaxin-1/CCL11 | -0.01 | 0.94 |
| RANTES/CCL5 | -0.12 | 0.29 |
| IL-5 | -0.05 | 0.65 |
| IL-6 | 0.02 | 0.85 |
| MIP-1β/CCL4 | 0.05 | 0.63 |
| VEGF | 0.18 | 0.14 |
| IL-8/CXCL-8 | 0.12 | 0.18 |
| IL1-β | 0.15 | 0.18 |
| MCP-1/CCL2 | 0.25 | 0.03 |
| IP-10/CXCL10 | 0.15 | 0.18 |

* Correlations performed on log_10_-transformed inflammatory marker concentrations.
